# Accessory pathway analysis using a multimodal deep learning model

**DOI:** 10.1038/s41598-021-87631-y

**Published:** 2021-04-13

**Authors:** Makoto Nishimori, Kunihiko Kiuchi, Kunihiro Nishimura, Kengo Kusano, Akihiro Yoshida, Kazumasa Adachi, Yasutaka Hirayama, Yuichiro Miyazaki, Ryudo Fujiwara, Philipp Sommer, Mustapha El Hamriti, Hiroshi Imada, Makoto Takemoto, Mitsuru Takami, Masakazu Shinohara, Ryuji Toh, Koji Fukuzawa, Ken-ichi Hirata

**Affiliations:** 1 Division of Cardiovascular Medicine, Department of Internal Medicine, Kobe University Hospital, 7-5-2, Kusunoki-Cho, Chuo-Ku, Kobe, Japan; 2 Preventive Medicine and Epidemiology, National Cerebral and Cardiovascular Center Research Institute, Suita, Japan; 3 Department of Cardiovascular Medicine, National Cerebral and Cardiovascular Center, Suita, Japan; 4 Kita-Harima Medical Center, Ono, Japan; 5 Akashi Medical Center, Akashi, Japan; 6 Saiseikai Nakatsu Hospital, Osaka, Japan; 7 Clinic of Electrophysiology, Heart and Diabetes Center NRW, University Hospital of Ruhr-University Bochum, Bochum, Germany; 8 Ako City Hospital, Ako, Japan; 9 Division of Epidemiology, Kobe University Graduate School of Medicine, Kobe, Japan; 10 Division of Evidence-Based Labolatory Medicine, Kobe University Graduate School of Medicine, Kobe, Japan

**Keywords:** Information technology, Cardiology

## Abstract

Cardiac accessory pathways (APs) in Wolff–Parkinson–White (WPW) syndrome are conventionally diagnosed with decision tree algorithms; however, there are problems with clinical usage. We assessed the efficacy of the artificial intelligence model using electrocardiography (ECG) and chest X-rays to identify the location of APs. We retrospectively used ECG and chest X-rays to analyse 206 patients with WPW syndrome. Each AP location was defined by an electrophysiological study and divided into four classifications. We developed a deep learning model to classify AP locations and compared the accuracy with that of conventional algorithms. Moreover, 1519 chest X-ray samples from other datasets were used for prior learning, and the combined chest X-ray image and ECG data were put into the previous model to evaluate whether the accuracy improved. The convolutional neural network (CNN) model using ECG data was significantly more accurate than the conventional tree algorithm. In the multimodal model, which implemented input from the combined ECG and chest X-ray data, the accuracy was significantly improved. Deep learning with a combination of ECG and chest X-ray data could effectively identify the AP location, which may be a novel deep learning model for a multimodal model.

## Introduction

Wolff–Parkinson–White (WPW) syndrome is caused by a congenital cardiac accessory pathway (AP) that can cause paroxysmal palpitations and occasionally fatal arrhythmias owing to complications involving atrial fibrillation^1,2^. In addition, as delta waves, which are characteristics of 12-lead electrocardiogram (ECG) waveforms obtained for patients with WPW syndrome, can be observed even in the absence of arrhythmia^3^, the diagnosis of WPW syndrome is relatively simple, while concealed WPW syndrome diagnosis is not.

Conventionally, a decision tree algorithm using a 12-lead ECG has been used to diagnose APs in WPW syndrome^4–6^. However, problems have been reported.

First, if the ECG is very ambiguous (e.g., when the polarity of the delta wave or QRS is exactly in the middle or when the electrical potential is too small to be judged), the decision-tree algorithm may not be able to provide a decision. Second, as conventional algorithms have been created by excluding atypical cases involving dual pathways and malformations^6^, those cases would always be classified into one of the categories that do not match them. Third, even if the polarity of the 12-lead ECG is the same, the location of the AP may subtly differ depending on the orientation and size of each heart. Owing to these problems, differences often exist between conventional and definitive results obtained by electrophysiological studies.

In recent years, artificial intelligence (AI) has rapidly developed in medicine. Various definitions of AI exist. One has recently attracted attention and is commonly known as deep learning, which is a machine learning model that uses multiple layers of neural networks. Extensive AI models based on deep learning have been developed, particularly for image classification. AI models corresponding to various modality images have been reported and include a model that classifies whether COVID-19 is present in the diagnosis of pneumonia by chest computed tomography^7^ and a model that identifies asynergy in echocardiography images^8^. In addition, deep learning can handle not only images but also time-series data, such as language and waveform data^9^.

Generally, deep learning requires a large amount of annotated data. It is relatively simple to collect data in authenticating ubiquitous objects, such as cars and human faces^10^, but the amount of medical data that we can collect is limited because of the limited number of patients. Notably, few medical AI models exist for practical use. In addition, because machine learning requires definitive answers together with training data, it cannot be applied to ambiguous cases that cannot be diagnosed, which hinders its practical use in medicine^11^.

To solve the abovementioned problems, we created a new model for the diagnosis of AP localization in WPW syndrome by using a medical deep learning model. The primary goal of this study was to make a model that was more accurate than a conventional algorithm. The secondary goal of this study was to resolve the problem that the location of an AP differs depending on the orientation and size of each heart, which can be solved by adding a chest X-ray image into the model so that the model can consider the axis or size of the heart.

## Methods

### Patients and data

**Figure 1:**
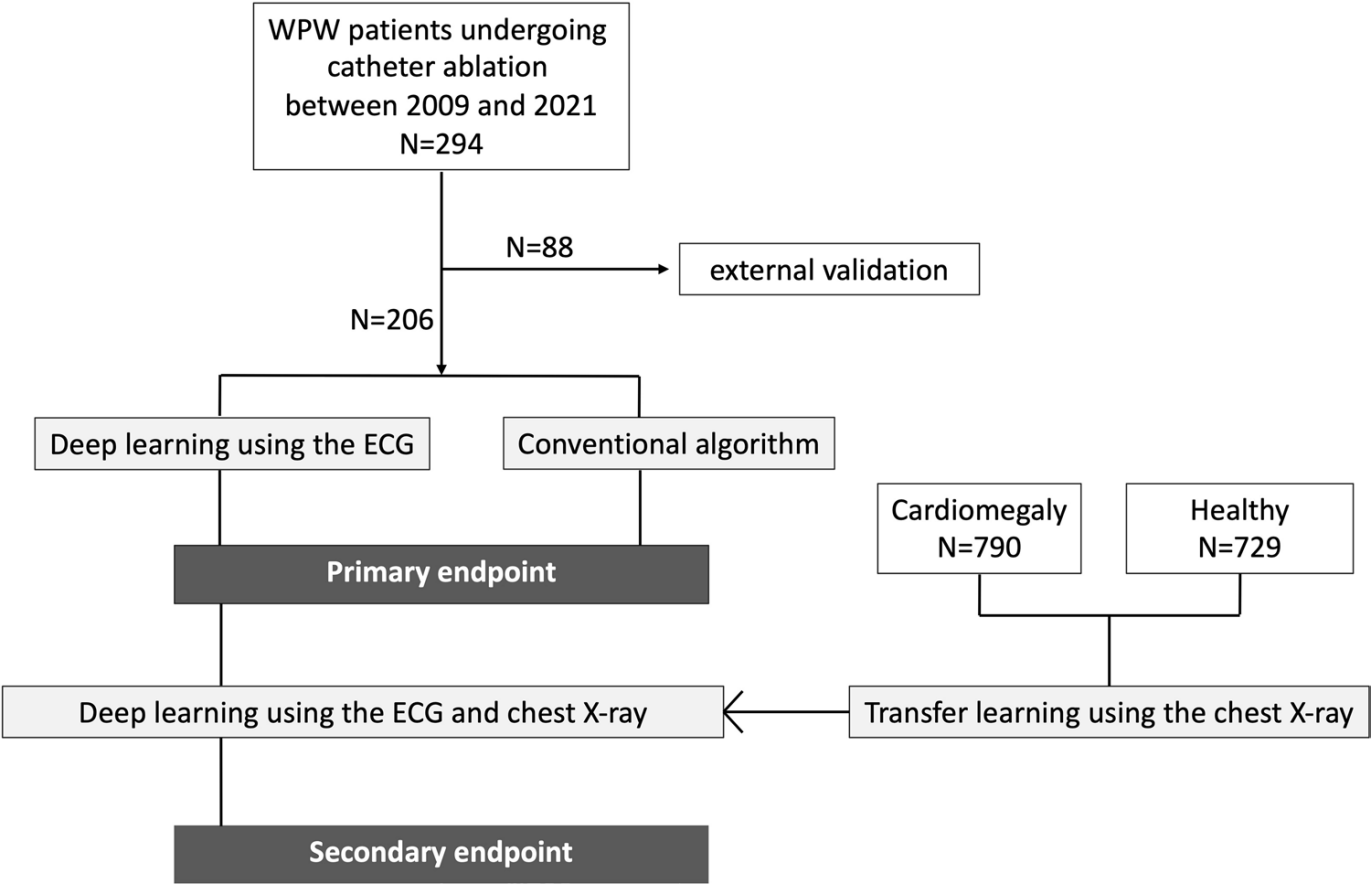
Study population and design. The primary endpoint was assessed by comparing the accuracy of each algorithm. The secondary endpoint was assessed by determining how the second model outcomes differed when the chest X-ray images were added into the model, rather than using only the ECG.

A total of 294 cases, including 240 cases with WPW syndrome and 54 normal cases from seven centres, were collected during the period from March 2009 to January 2021 (Fig. 1). A total of 206 cases collected from six centres were used as the training dataset and internal validation dataset, and a total of 88 cases collected from another centre were used as the external validation dataset. We collected preoperative 12-lead ECG data and preoperative chest X-ray data from patients who underwent ablation therapy for WPW syndrome. The chest X-ray data were acquired from posterior to anterior. Patients with atrioventricular reentrant tachycardia whose delta waves could not be observed during normal sinus rhythm were excluded from this study. All methods were carried out in accordance with the relevant directives and regulations, as well as the Declaration of Helsinki, and informed consent was obtained from all the participants of the experiments. This clinical study was approved by the ethical review boards of Kobe University Medical Ethical Committee (No. 190164) on November 1, 2019.

### Electrophysiology studies

An electrophysiological study and catheter ablation were performed in a total of 174 patients. After precise mapping was performed without or with saline irrigation using a 7-Fr or 8-Fr deflectable catheter with a 4- or 3.5-mm tip and an interelectrode spacing of 2.5 or 2 mm, a radiofrequency current was delivered. Left-sided APs were approached from the ventricular aspect of the mitral annulus through the aorta or from the atrial aspect of the mitral annulus through the atrial septum. Right-sided APs were approached by positioning the ablation catheter on the tricuspid valve annulus via the femoral vein. Posteroseptal APs were approached by positioning the ablation catheter on the medial aspect of the mitral annulus through a retrograde transaortic route, on the tricuspid annulus, or within the proximal coronary sinus via the femoral vein.

**Figure 2:**
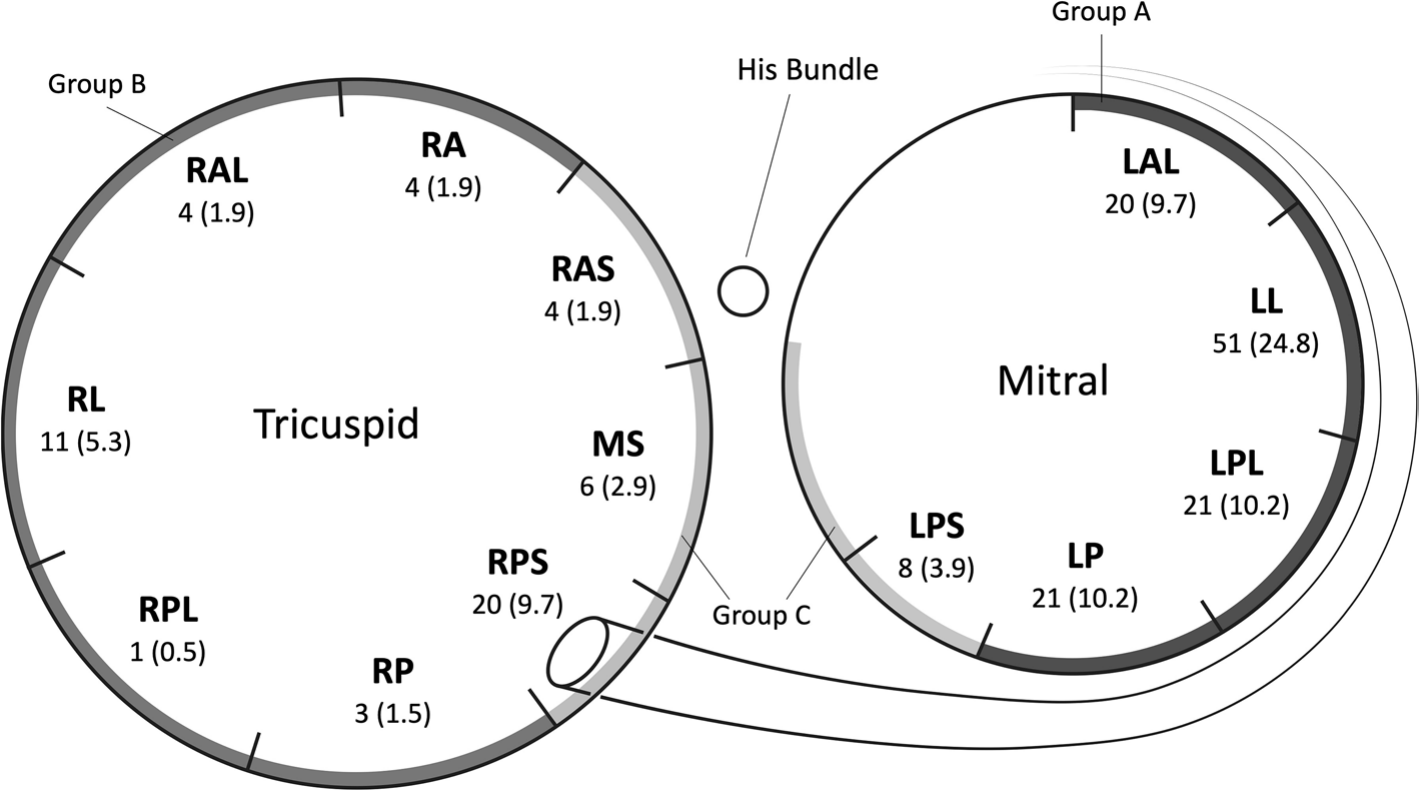
Accessory pathway locations. The numbers of samples (proportions) are shown at each location. *RAS* right anteroseptal, *RA* right anterior, *RAL* right anterolateral, *RL* right lateral, *RPL* right posterolateral, *RP* right posterior, *RPS* right posteroseptal, *MS* right and left mid septal, *LAL* left anterolateral, *LL* left lateral, *LPL* left posterolateral, *LP* left posterior, *LPS* left posteroseptal.

A standard pre-excited 12-lead ECG was obtained with simultaneous recordings on three channels during spontaneous sinus rhythm before the electrophysiological study and radiofrequency catheter ablation. The recording speed was 25 mm/s, while the amplification was 10 mm/mV. The polarity of the QRS complexes (positive, negative, or equiphasic), morphology of the QRS complexes, and highest R-wave amplitude in the precordial leads were analysed. Thirteen regions were identified along the atrioventricular annuli according to the results of catheter mapping during ablation (right anteroseptal [RAS], right anterior [RA], right lateral [RL], right anterolateral [RAL], right posterolateral [RPL], right posterior [RP], right posteroseptal [RPS], right and left mid septal [MS], left anterolateral [LAL], left lateral [LL], left posterolateral [LPL], left posterior [LP], and left posteroseptal [LPS] regions) (Fig. 2). To simplify the classification, LAL, LL, LPL, and LP were defined as Group A, RA, RP, RPL, RL, and RAL were defined as Group B, RAS, LPS, MS, and RPS were defined as Group C, and normal cases were defined as Group N, which led to four classifications.

### Data preprocessing

The ECG data for each of the 12 leads were converted to one-dimensional data to reduce the calculation cost. The ECG data were collected from multiple facilities as image data; we subsequently cropped the image into separate two-dimensional image data, comprising one heartbeat for each lead. Each heartbeat was cropped to a 250 × 375-pixel image, with 4.8 ms and 2.4 µV set as one pixel. Finally, we converted each lead image into one-dimensional data, i.e., we converted the voltage at each time point into a single number. The final matrix shape is (n, 250, 12), where n is the number of samples, 250 is the number of time points, and 12 is the number of leads. The chest X-ray data were compressed and cropped to 200 × 200 pixels, as the calculation cost would be large if the image size were too large to input into the model.

As we needed to investigate the generalization performance with a small number of samples, we verified the generalization performance of the AI model using the five-fold cross-validation method^12^ and calculated the average accuracy and loss.

### Development of deep learning models

In this study, we used a convolutional neural network (CNN) model^13^, widely used in image recognition, as the main deep learning model. Among the various CNN models, a one-dimensional CNN model was used to input the ECG data. As the input data lengths and number of samples were small, a relatively small model was created. Overall, the network model contained 16 convolution layers, followed by a fully connected layer and softmax layer, which calculated the probability of each of the four as the output in the last layer (Supplementary Table 1). We used the Adamax optimizer with the default parameters β1 = 0.9, β2 = 0.999, and a mini-batch size of 32. We chose the model that achieved the lowest error in the derivation dataset. The hyperparameters of the algorithm architecture and optimisation algorithm were selected using Bayesian optimisation^14^.

**Figure 3:**
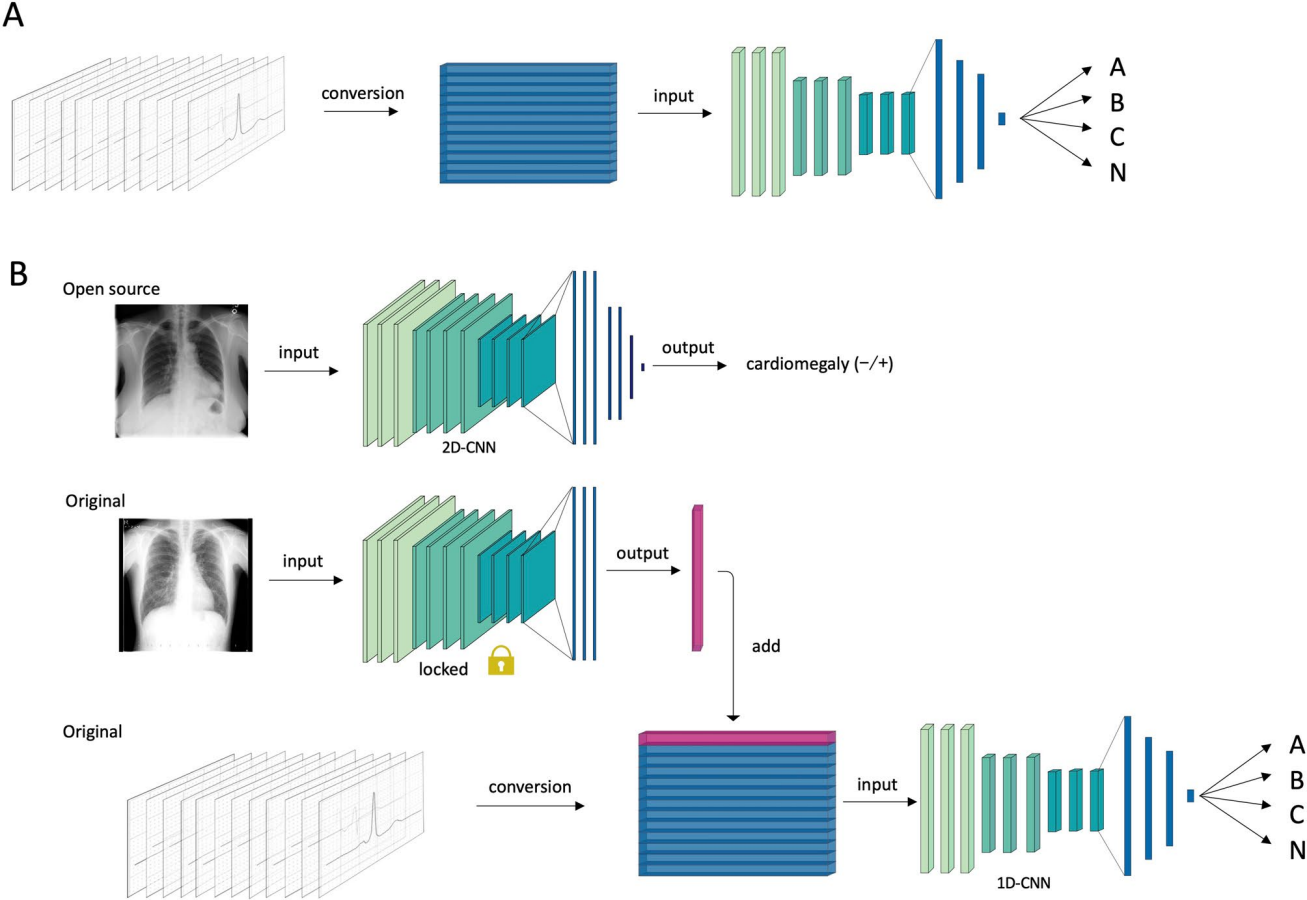
Overview of the deep learning architecture. (**A**) For the primary output, 206 ECGs were loaded into the convolutional neural network model and classified into four classifications (Group A, Group B, Group C, and Group N [normal class]). (**B**) For the secondary output, chest X-ray images from 1519 patients were used for pretraining, and the original chest X-ray images were compressed into a one-dimensional vector using the pretrained midlayer weights. The combined ECG and chest X-ray data were finally trained to classify the outputs. 2D-CNN indicates a two-dimensional convolutional neural network, and 1D-CNN indicates a one-dimensional convolutional neural network.

For the learning of chest X-ray data, we used a two-dimensional CNN model in three phases (Fig. 3). First, we used the ResNet50 model^15^, which has residual blocks and trains extremely deep neural networks to perform transfer learning using another chest X-ray dataset. We used 1519 open-source chest X-ray images provided by the National Institutes of Health (NIH) Clinical Center, including images from cardiomegaly and normal cases, and trained the Resnet50 model to classify whether cardiac enlargement was present. We then input our original chest X-ray dataset into the well-trained Resnet50 model, obtained the outputs of the trained Resnet50 model, which contained highly dimensional compressed chest X-ray information, and applied it to the next phase.

Second, we reshaped the outputs of the ResNet50 model into the same shape as that of the single-lead ECG data so that we could combine the outputs of the ECG model with those of the ResNet50 model. Thus, the concatenated output had a size of 13 ECG leads.

Finally, we trained the combined data with 16 layers of the neural network and predicted the AP classification. To evaluate whether the addition of X-ray data to the model improved the statistical results, we input an empty layer instead of compressed X-ray image data into the model so that we could fairly compare the statistical results. The positive predictive value (PPV), sensitivity, and F1-score were calculated in addition to the accuracy. Each statistic was compared using a t-test.

For external validation, we collected an additional 88 samples from another centre. After learning with the training dataset and cross-validation with the internal validation dataset, the weights of the model were locked in, and the location was predicted using an external validation dataset. The accuracy, PPV, sensitivity, and micro F1-score of the conventional algorithm, the deep learning model using only ECG data, and the deep learning model using both ECG and chest X-ray data were compared for overall samples.

For the construction of this machine learning model, the programming language Python and TensorFlow framework^16^ were used.

### Data augmentation

Data augmentation is a strategy that can significantly increase the diversity of data available for training models without actually collecting new data. We applied data augmentation techniques to the ECG and chest X-ray data. Refer to the supplemental material (Supplementary Fig. 1) for further details.

## Results

### Patient characteristics and successful ablation site of APs

Among the participants, 135 were male (112 WPW syndrome cases), while 71 were female (62 WPW syndrome cases). The average [SD] age was 46.9 [17.5] years (range 13–84 years). The AP proportions of all 174 cases and each group are shown in Table 1. The ratios of the classifications to the entire sample were 113 (54.9%) for Group A, 23 (11.2%) for Group B, 38 (18.4%) for Group C, and 32 (15.5%) for Group N.

**Table 1:** Classifications of the accessory pathway locations.

	No. (%)
Group A	113 (54.9)
LAL	20 (9.7)
LL	51 (24.8)
LP	21 (10.2)
LPL	21 (10.2)
Group B	23 (11.2)
RA	4 (1.9)
RAL	4 (1.9)
RL	11 (5.3)
RP	3 (1.5)
RPL	1 (0.5)
Group C	38 (18.4)
LPS	8 (3.9)
MS	6 (2.9)
RAS	4 (1.9)
RPS	20 (9.7)
Group N	32 (15.5)
Total	206

Accessory pathway locations and proportions.

*LAL* left anterolateral, *LL* left lateral, *LP* left posterior, *LPL* left posterolateral, *LPS* left posteroseptal, *MS* right and left mid septal, *RA* right anterior, *RAL* right anterolateral, *RAS* right anteroseptal, *RL* right lateral, *RP* right posterior, *RPL* right posterolateral, *RPS* right posteroseptal. LAL, LL, LPL, and LP were defined as Group A, RA, RP, RPL, RL, and RAL were defined as Group B, RAS, LPS, MS and RPS were defined as Group C, and normal cases were defined as Group N.

### Comparison to the conventional algorithm

We also applied the conventional algorithm (the St George's decision tree algorithm^6^) to the ECG dataset to compare its accuracy to that of our deep learning model in the diagnosis of AP locations on the 12-lead ECG. The tree algorithm is shown in Supplementary Fig. 2. The PPV, sensitivity, and F1-score of the conventional algorithm and deep learning model using only the ECG for each group (Group A, Group B, and Group C) are shown in Table 2. The statistics of the deep learning model using only the ECG are shown in the ‘ECG’ column. The accuracy of the conventional algorithm was 0.61. On the other hand, the PPV, sensitivity, and F1-score of the deep learning model for Group A and Group B were significantly higher than those of the conventional algorithm (p < 0.001; one-sample t-test), and the PPV of the deep learning model for Group C was also significantly higher. The mean [SD] accuracy of the deep learning model was 0.78 [0.02], which was significantly higher than that of the conventional algorithm (p < 0.001; one-sample t-test).

**Table 2:** Statistics for each algorithm.

		Group N	Group A	Group B	Group C
Conventional	PPV	–	0.81	0.29	0.40
	Sensitivity	–	0.71	0.39	0.46
	F1-score	–	0.76	0.33	0.43
	Accuracy	0.61			
ECG	PPV, mean (SD)	0.78 (0.04)	0.90 (0.01)	0.56 (0.05)	0.49 (0.02)
	p value*	–	< 0.001	< 0.001	< 0.001
	Sensitivity, mean (SD)	0.97 (0.01)	0.86 (0.02)	0.61 (0.04)	0.40 (0.04)
	p value*	–	< 0.001	< 0.001	0.84
	F1-score, mean (SD)	0.86 (0.02)	0.88 (0.02)	0.58 (0.04)	0.44 (0.03)
	p value*	–	< 0.001	< 0.001	0.019
	Accuracy, mean (SD)	0.78 (0.02)			
	p value*	< 0.001			
ECG and X-ray	PPV, mean (SD)	0.80 (0.04)	0.91 (0.02)	0.63 (0.05)	0.58 (0.06)
	p value^+^	0.44	0.23	0.09	0.06
	Sensitivity, mean (SD)	0.99 (0.02)	0.88 (0.02)	0.73 (0.04)	0.40 (0.07)
	p value^+^	0.10	0.22	0.0011	0.89
	F1-score, mean (SD)	0.88 (0.03)	0.89 (0.01)	0.67 (0.05)	0.46 (0.07)
	p value^+^	0.23	0.24	0.008	0.64
	Accuracy, mean (SD)	0.80 (0.02)			
	p value^+^	0.040			

We analysed the performance of each model. The conventional model was performed once, and the ‘ECG’ and ‘ECG and X-ray’ model was repeatedly performed to analyse the statistics. PPV indicates the positive predictive value; ECG is the abbreviation for echocardiogram. Data are expressed as the mean (standard deviation). The *p value compares the statistics of the conventional algorithm and the deep learning model using only ECG data. The ^+^p value compares the statistics of the deep learning model using only ECGs and the model using both ECG and chest X-ray data.

### Deep learning model with chest X-ray and ECG data

In the multimodal model, which implemented the input of the combined ECG and chest X-ray data, the mean accuracy was 0.80 [0.02], which was significantly higher than that of the model using only the ECG data (p < 0.05). The PPV, sensitivity, and F1-score for each group are also shown in Table 2, and these statistics are shown in the ‘ECG and X-ray’ column. The sensitivity of the multimodal model for Group B significantly improved from that of the deep learning model using only the ECG data (p < 0.01), and the F1-score of the multimodal model for Group B also significantly improved (p < 0.05). The accuracy and loss of each deep learning model are depicted in Supplementary Fig. 3a,b, respectively.

### External validation

For external validation, we identified 88 individuals from another facility. Among the participants, 56 were male (40 WPW syndrome cases) and 32 were female (26 WPW syndrome cases). The ratios of the classifications to the entire sample were 45 (51.1%) for Group A, 13 (14.7%) for Group B, 8 (9.0%) for Group C, and 22 (25.0%) for Group N. The overall accuracy, weighted PPV, weighted sensitivity, and weighted F1-score of the deep learning model with the external validation dataset are shown in Supplementary Table 2. The deep learning model using only ECG data was significantly more accurate than the conventional algorithm (p < 0.001), and the deep learning model using both ECG and chest X-ray data was significantly more accurate than the deep learning model using only ECG data (p = 0.044).

### Representative case

A representative ECG where the AP was located in the left posterolateral region is shown in Supplementary Fig. 4. In this case, the conventional algorithm predicted that the location was in the right anteroseptal region, while the multimodal deep learning model correctly predicted the location as Group A.

## Discussion

### Main findings

In this study, we used 12-lead ECG data from patients with and without WPW syndrome to create a machine learning model using a one-dimensional CNN, which differs from the conventional decision tree algorithm. In addition, another machine learning model inputting data with two different modalities was created using the transfer learning method, which further improved the accuracy. It is important to accurately predict the location of the accessory pathway in a clinical setting because location prediction affects acute and chronic success rates of treatment. For accurate prediction, we need a method that is unaffected by the reader or orientation of the heart. Therefore, the proposed diagnostic model can be useful for AP classification in WPW syndrome.

### Advantages of the deep learning model

The conventional method using the decision tree algorithm had two problems. First, if the polarity of the delta wave or QRS wave could not be determined because of an ambiguous waveform, it was impossible to achieve a true diagnosis. The conventional method depends on the subjectivity and experience of the person who makes the judgement. If the undecidable judgement is on the initial branch of the decision tree, the algorithm would lead to large errors, whereas a neural network model could stochastically provide an overall judgement. Thus, a neural network algorithm might be a more robust algorithm than a decision tree algorithm.

Second, the conventional method could not predict the probability of the classification, whereas in the case of a neural network, the probability of each classification could be calculated. Therefore, if the probability of any classification is generally low, it can be classified as atypical. Numerous atypical cases, such as dual APs, skewed APs, or anomaly cases, exist in clinical practice^17^. It would be useful to evaluate whether the case is atypical and explain such information to the patient before surgery. Although we could not obtain sufficient data from atypical cases, in theory, it would be possible to predict whether the case was atypical.

It is important to accurately predict the location of the accessory pathway in a clinical setting because location prediction affects the acute and chronic success rate of treatment. In particular, treatment of the posteroseptal and right-side accessory pathways has a poorer outcome than the left lateral accessory pathway due to anatomical reasons^17^. Therefore, accurate prediction is important in developing an ablation strategy (e.g., selecting an approach method or selecting the type of ablation sheath). For accurate prediction, we developed a method that is unaffected by the reader or rotation of the heart, using deep learning.

### Model learning with small samples

Deep learning is used in various applications, such as face recognition^18^ and self-driving techniques^19^, where a very large amount of data needs to be learned. However, in clinical settings, it could be challenging to obtain sufficient medical data because of strict privacy policies and lack of interhospital data sharing systems. In the case of small set of medical data, including images and other clinical information, transfer learning techniques will be effective in achieving good accuracies with fewer labelled datasets.

Transfer learning enables the transfer of knowledge learned from one dataset to another, which can lead to a reduction in the learning time and improvement in the accuracy, even when the amount of data is small^20^. We extracted the features of the patient's chest X-ray images from the middle of the layers of the pretrained model, as depicted in Fig. 3. By this process, we could transform the 200 × 200 × 3-dimensional data to 250-dimensional data, which still had information on the size and orientation of the heart, even in smaller dimensional data. The purpose of using cardiomegaly annotation in the pretraining phase was to create a pretrained model that could extract features from chest X-ray images by focusing on the size and orientation of the heart.

In general, the dimensions of each sample (e.g., the number of dots that the image has) can affect the performance of the machine learning model^21^. The higher the number of dimensions, the larger the number of samples needed. In addition, noisy data also require many samples. Therefore, it is necessary to use high-quality and low-dimensional data in learning with few samples. In this respect, as the ECG is a standardized modality and has little redundant data, its quality is higher than that of other data such as face images and voice data. Moreover, because the ECG is sequential wave data, the number of dimensions per sample is relatively small. Thus, effective learning is possible, even with a small sample size.

### Addition of X-ray image improving the accuracy

There is no algorithm for achieving a completely accurate diagnosis using only the ECG because the location may differ subtly depending on the orientation, rotation, and size of each heart, even if the polarity of the 12-lead ECG is the same. In general diagnostic routines before ablation treatment, only electrical information is used to determine the AP location, but we obtained better accuracy by adding the chest X-ray data to the electrical information, probably because the anatomical position information of the chest X-ray was used to correct the slightly different heart orientations of the individual. Given that adding anatomical information could improve accuracy, using the lateral view in addition to the frontal view of the chest X-ray or using computed tomography may produce a better outcome, which is the next step of our study.

Although a multimodal model is an ideal AI model in medicine, it might be more difficult to develop a multimodal model than a single modal model. Clinicians rarely provide diagnoses based on a single examination, but often, multiple examinations and background data of individual patients are used. Therefore, the multimodal model would be considered an ideal deep learning model. However, multiple inputs of various kinds of data cause problems of unexpected learning imbalances between the multiple inputs, and the model cannot be effectively trained. Considering that the ECG was more important than the X-ray image in the specification of the AP position, the X-ray images were compressed to the same size as a single lead of the ECG, so we could learn the ECG data more intensively.

### Limitations

As a limitation of this study, the number of classifications was only three, which may not be sufficient for practical applications. Although it would be better to apply all AP classifications, too few samples were used to efficiently learn all AP classifications, including a single sample, such as the RPL region. However, these three classifications were based on the previous reports^22,23^ and had a clinical significance such as the choice of the ablation strategy. Even with the use of data augmentation and transfer learning methods in the three categories, the F1-scores in Group B and Group C were still relatively low due to the small number of cases in our study. These problems could be solved if a further increase in the number of samples is provided in the future.

## Conclusions

A deep learning model based on 1D-CNN using ECG waveforms more accurately identified the AP location than the conventional algorithm. Moreover, the addition of chest X-ray images into the model to create a novel deep learning model, such as a multimodal model, could significantly improve the accuracy.

## Supplementary Information

Supplementary Information.

## Data Availability

All data collected for the study are not publicly available due to regulation from the Ethics Committee of the Kobe University and other institutions but are available from the corresponding author on reasonable request. Open source data of chest X-rays are available in the repository of National Institutes of Health—Clinical Center (https://nihcc.app.box.com/v/ChestXray-NIHCC).
