# Supplementary material for: Accessory pathway analysis using a multimodal deep learning model
Source: Sci Rep. 2021 Apr 13;11:8045. doi: 10.1038/s41598-021-87631-y (PMC8044112; doi:10.1038/s41598-021-87631-y)
Supplement: Supplementary file 1 — Supplementary Information. [file 41598_2021_87631_MOESM1_ESM.docx]

Accessory pathway analysis using a multimodal deep learning model

Makoto Nishimori^1^ (MD), Kunihiko Kiuchi^1^ (MD, PhD), Kunihiro Nishimura^2^ (MD, PhD), Kengo Kusano^3^ (MD, PhD), Akihiro Yoshida^4^ (MD, PhD), Kazumasa Adachi^5^ (MD, PhD), Yasutaka Hirayama^5^ (MD), Yuichiro Miyazaki^5^ (MD), Ryudo Fujiwara^6^ (MD, PhD), Philipp Sommer^7^ (MD, PhD), Mustapha El Hamriti^7^(MD), Hiroshi Imada^8^ (MD, PhD), Makoto Takemoto^1^ (MD), Mitsuru Takami^1^ (MD, PhD), Masakazu Shinohara^9^ (MD, PhD), Ryuji Toh^10^ (MD, PhD), Koji Fukuzawa^1^(MD, PhD), Ken-ichi Hirata^1^(MD, PhD)

1. Division of Cardiovascular Medicine, Department of Internal Medicine, Kobe University Hospital

2. Preventive Medicine and Epidemiology, National Cerebral and Cardiovascular Center Research Institute

3. Department of Cardiovascular Medicine, National Cerebral and Cardiovascular Center

4. Kita-harima Medical Center

5. Akashi Medical Center

6. Saiseikai Nakatsu Hospital

7. Clinic of Electrophysiology, Heart and Diabetes Center NRW, University Hospital of Ruhr-University Bochum

8. Ako City Hospital

9. Division of Epidemiology, Kobe University Graduate School of Medicine

10. Division of Evidence-based Labolatory Medicine, Kobe University Graduate School of Medicine**SUPPLEMENTAL MATERIAL**

**Sup. Table 1.** Basic deep learning model architecture

| Layer | Type | Kernel × Filter | Other Parameters |
| --- | --- | --- | --- |
| 1 | 1D-Convolutioin | 3 × 12 | Padding = 'same', Strides = 1 |
| 2 | Relu Activation |  |  |
| 3 | Batch Normalization |  |  |
| 4 | 1D-MaxPooling |  | Size = 2 |
| 5 | 1D-Convolutioin | 3 × 16 | Padding = 'same', Strides = 1 |
| 6 | Relu Activation |  |  |
| 7 | Batch Normalization |  |  |
| 8 | 1D-MaxPooling |  | Size = 2 |
| 9 | 1D-Convolutioin | 3 × 32 | Padding = 'same', Strides = 1 |
| 10 | Relu Activation |  |  |
| 11 | Batch Normalization |  |  |
| 12 | 1D-MaxPooling |  | Size = 2 |
| 13 | 1D-Convolutioin | 3 × 64 | Padding = 'same', Strides = 1 |
| 14 | Relu Activation |  |  |
| 15 | Batch Normalization |  |  |
| 16 | 1D-MaxPooling |  | Size = 2 |
| 17 | Flatten |  |  |
| 18 | Dense | 1 × 64 |  |
| 19 | Relu Activation |  |  |
| 20 | Batch Normalization |  |  |
| 21 | Dropout |  | Rate = 0.4 |
| 22 | Dense (output) | 1 × 4 | Activation = 'softmax' |

The basic one-dimensional convolutional neural network (1D-CNN) model contained 16 convolution layers, followed by a fully connected layer, Dense layer, and softmax layer, which calculated the probability of each of the four as the output in the last layer.

**Sup. Table 2.** Statistics for each algorithm

| model | metric |  |
| --- | --- | --- |
| Conventional | PPV | 0.7 |
|  | Sensitivity | 0.59 |
|  | F1-score | 0.61 |
|  | Accuracy | 0.59 |
| ECG | PPV, mean (SD) | 0.74 (0.04) |
|  | p value* | 0.010 |
|  | Sensitivity, mean (SD) | 0.73 (0.04) |
|  | p value* | <0.001 |
|  | F1-score, mean (SD) | 0.73 (0.04) |
|  | p value* | <0.001 |
|  | Accuracy, mean (SD) | 0.73 (0.04) |
|  | p value* | <0.001 |
| ECG and X-ray | PPV, mean (SD) | 0.77 (0.02) |
|  | p value^+^ | 0.094 |
|  | Sensitivity, mean (SD) | 0.76 (0.02) |
|  | p value^+^ | 0.044 |
|  | F1-score, mean (SD) | 0.75 (0.03) |
|  | p value^+^ | 0.19 |
|  | Accuracy, mean (SD) | 0.76 (0.02) |
|  | p value^+^ | 0.044 |

We used an external validation dataset to analyse the performance of both the ‘ECG’ and the ‘ECG and X-ray’ models. Each model was repeatedly used to analyse the statistics. PPV indicates positive predictive value; ECG indicates echocardiogram. Data are expressed as the mean (standard deviation). The *p value compares each of the statistics of the conventional algorithm and the deep learning model using only ECG data. The ^+^p value compares each of the statistics of the deep learning model using only ECG data and the model using both ECG and chest X-ray data.

**Sup. Figure 1.** Data augmentation for the ECG and chest X-ray data


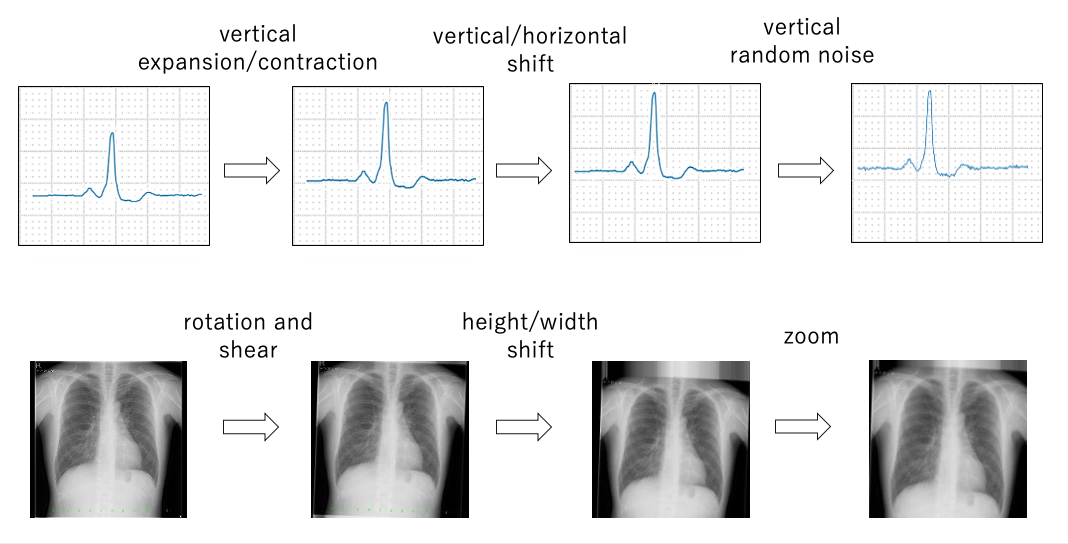


Each type of data was augmented in three steps. All variations of modification were independently and randomly executed in each training epoch. The method of augmentation performed on the ECG included vertical expansion/contraction (up to 10%), vertical translation (up to 10 px), horizontal translation (up to 20 px), and vertical random noise (up to 3 px). In addition, the chest X-ray image was augmented using rotation (up to 20°), shear (up to 5°), height shift (up to 10%), and width shift (up to 10%). These parameters were randomly selected in the indicated ranges. The augmentation methods described above were randomly applied for each learning epoch. Exactly the same data were learned only once during all learning phases, which created a robust and versatile model against noise and individual variations. Another augmentation method, such as vertical or horizontal flipping, is commonly used; however, horizontal or vertical flipping is not effective in our cases because ECGs and chest X-rays are not symmetric. Therefore, the methods of augmentation should be selected according to each modality.

**Sup. Figure 2.** The St George's tree algorithm

St George’s algorithm for the localization of accessory pathways. +ve = QRS complex-positive; -ve = QRS complex-negative; +/-ve = QRS complex-equiphasic; AP = accessory pathway; LAL = left anterolateral; LP = left posterior; LPL = left posterolateral; LPS = left posteroseptal; MS = mid-septal; RAS = right anteroseptal; RL = right lateral; RP = right posterior; and RPS = right posteroseptal.

**Sup.Figure 3.** Outcomes for predicting accessory pathways

****Comparison of the accuracies (A) and losses (B) of the model using only echocardiogram data (blue line) and the model using echocardiogram data combined with chest X-ray data (green line) for predicting accessory pathways. The translucent bands indicate the 95% confidence intervals.

**Sup. Figure 4** The ECG of a representative case


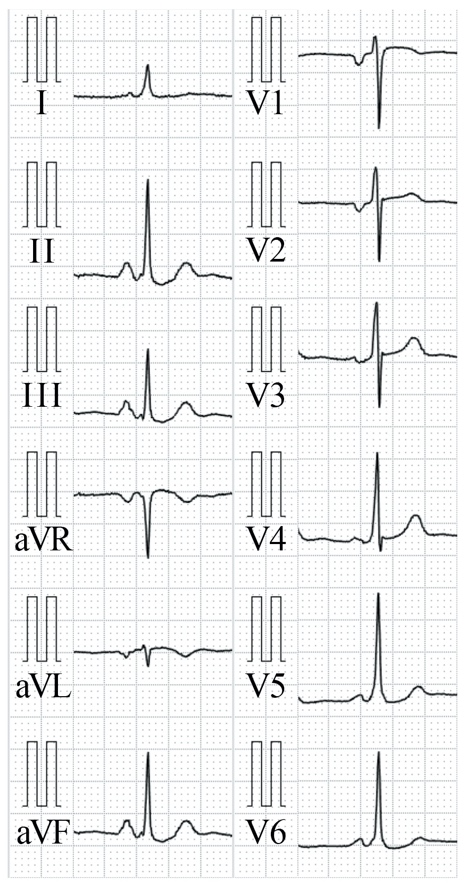
A representative case of WPW syndrome where the accessory pathway was located in the left posterolateral region. In this case, the conventional algorithm predicted that the location was in the right anteroseptal region, while the multimodal deep learning model correctly predicted the location as Group A.
